# Executive Function and IQ Predict Mathematical and Attention Problems in Very Preterm Children

**DOI:** 10.1371/journal.pone.0055994

**Published:** 2013-02-04

**Authors:** Cornelieke Sandrine Hanan Aarnoudse-Moens, Nynke Weisglas-Kuperus, Hugo Joseph Duivenvoorden, Johannes Bernard van Goudoever, Jaap Oosterlaan

**Affiliations:** 1 Department of Paediatrics, Erasmus University Medical Centre, Rotterdam, The Netherlands; 2 Department of Paediatrics, Amsterdam Medical Centre, Amsterdam, The Netherlands; 3 Department of Paediatrics, VU University Medical Centre, Amsterdam, The Netherlands; 4 Department of Clinical Neuropsychology, VU University Amsterdam, Amsterdam, The Netherlands; University of Granada, Spain

## Abstract

Objective of this study was to examine the impact of executive function (EF) on mathematical and attention problems in very preterm (gestational age ≤ 30 weeks) children. Participants were 200 very preterm (mean age 8.2 ± 2.5 years) and 230 term children (mean age 8.3 ± 2.3 years) without severe disabilities, born between 1996 and 2004. EFs assessed included verbal fluency, verbal working memory, visuospatial span, planning, and impulse control. Mathematics was assessed with the Dutch Pupil Monitoring System and parents and teachers rated attention problems using standardized behavior questionnaires. The impact of EF was calculated over and above processing speed indices and IQ. Interactions with group (very preterm versus term birth status) were examined. Analyses were conducted separately for two subsamples: children in preschool and children in primary school. Very preterm children performed poorer on tests for mathematics and had more parent and teacher rated attention problems than term controls (ß_s_>.11, P_s_<.01). IQ contributed unique variance to mathematics in preschool and in primary school (ß_s_>.16, P_s_<.007). A significant interaction of group with IQ (ß = −. 24, P = .02) showed that IQ contributed unique variance to attention problems as rated by teachers, but that effects were stronger for very preterm than for term infants. Over and above IQ, EF contributed unique variance to mathematics in primary school (ß = .13, P<.001), to parent rated inattention in preschool and in primary school (ß_s_>−.16, P_s_<.04), and to teacher rated inattention in primary school (ß = −.19; ß = .19, P_s_<.009). In conclusion, impaired EF is, over and above impaired IQ, an important predictor for poor mathematics and attention problems following very preterm birth.

## Introduction

Most very preterm (gestational age ≤ 30 weeks) infants survive without major disabilities.[1] However, a majority of these 'non-disabled' survivors suffer from academic and behavior problems that persist into adulthood.[2] About 70% of this population has special educational needs, and the social and economic burden is large. The most pronounced academic and behavior problems are poor mathematics and attention problems.[3], [4] We have recently shown that preschool mathematical abilities comprising numerical reasoning skills are already substantially impaired in very preterm children.[5] To enable early intervention, more insight in mechanisms involved in these mathematical and attentional problems is needed.

A large body of literature on term children has demonstrated that higher-order neurocognitive processes, the so-called executive functions (EF) are crucial in explaining academic difficulties and behavior problems.[6]–[13] EF are prefrontal brain functions that control thought and behavior. Typical lists of EF include the capacity to mentally manipulate information in mind (i.e. working memory), generating as many different solutions for a particular problem as possible (i.e. fluency), developing strategies to reach a future goal (i.e. planning), and inhibiting responses to irrelevant stimuli (i.e. impulse control).[10], [14], [15]

Research has consistently described that very preterm birth affects EF.[3], [16]–[18] Nevertheless, studies linking impaired EF to academic achievement and behavioral difficulties in this population remain scarce.[19]–[23] The few available studies have shown that very preterm children's poor impulse control and working memory skills are related to academic underperformance and inattentive behavior. Some studies, however, have suggested that slow processing speed underlies this relationship.[20], [22] Slowed processing speed results from white matter abnormalities,[24] a phenomenon frequently observed in very preterm children.[25]–[27] Compromised white matter may as well result in inconsistent speed, [28] with major trial-to-trial variations in performance. Such variations have been postulated as the specific deficiency in Attention Deficit Hyperactivity Disorder.[29]–[31] and may as well characterize attention problems in the very preterm population.[32]

Given the ongoing debate on whether IQ and EF are related or distinct concepts,[33]–[35] earlier studies have compared the impact of impaired EF on poor academic and behavioral outcomes in very preterm children to that of impaired IQ.[23] However, poor performance on IQ tests might be caused by impaired executive processes[33], and assessment of IQ may not fully capture the range of impaired neurocognitive skills underlying poor academic and behavioral outcomes in very preterm children.[36]

Aim of this study was to capture the unique contribution of EF to mathematical and attention problems in very preterm children over and above that of processing speed indices and IQ. EF’s were selected on which our very preterm sample had deficits [>.3 standardized mean difference [SMD]) compared to term control children.[18] Analyses were performed on a large sample of very preterm and term control children aged 4 to 12 years who were either in preschool or in primary school. Very preterm and term children were comparable in age and sex and free of severe disabilities.

## Methods

### Participants

The sample of 200 very preterm (gestational age ≤ 30 weeks) children was derived from all (n = 1260) very preterm infants admitted between 1996–2004 to the neonatal intensive care unit (NICU) of the Erasmus University Medical Centre, Sophia Children's Hospital Rotterdam, The Netherlands. 252 infants died. Twins (n = 302) were excluded as inclusion of these children would violate the assumption of independence of data. Very preterm children with severe disabilities not being able to perform tests as employed in the present study were also excluded (n = 77). Such severe disabilities were classified according to Wood et al,[37] which defined a severe disability as one that was likely to put the child in need of physical assistance to perform daily activities.[37] These children were excluded on the basis of their medical records. Remaining children were traced and if possible invited to participate (n = 270). Parents of 70 children were not willing to participate. There were no significant differences with respect to gestational age, birthweight, duration of NICU- stay between the included year cohorts (each year cohort was compared with all other year cohorts, all F_s_<.8; all P_s_>.6). The term control group (*n* = 230) was recruited from three regular schools located in the same neighbourhoods as schools attended by the very preterm children, and included children without histories of prematurity (gestational age > 37 wk), perinatal complications, and neurological disorders. The present study was carried out in the years 2007 and 2008. Parents of all participating children provided informed consent. The medical ethics review board of the Erasmus University Medical Centre Rotterdam approved the study protocol.

Minor neurosensory dysfunctions as observed in participating children are presented in Table 1( and included 1) vision corrected to normal with contact lenses or glasses, (2) hearing loss corrected to normal with hearing aids, (3) spastic unilateral cerebral palsy classified according to standards of the Surveillance of Cerebral Palsy in Europe (SCPE, 2000).

**Table 1:** Sample Characteristics of the Very Preterm and Term Group.

	Groups					
	Very Preterm (n = 200)			Term (n = 230)		
Age^a^, mean, SD, range, y	8.2	2.5	4.0–12.0	8.3	2.3	4.0–12.0
Gestational Age, mean, SD, range, wk	28.1	1.4	24.5–30.0	39.9	1.2	37.0–43.0
<28 wk, n, %	87.0	43.5		0.0	0.0	
Birthweight, mean, SD, range, g	1013.0	287.0	460.0–1900.0	3578.0	482.0	2500.0–5025.0
<1500 g, n, %	191.0	95.5		0.0	0.0	
Boys, n, %	106.0	53.0		106.0	46.1	
Estimated IQ^b^	93.3	15.8	70.0–138.0	105.0	13.4	70.0–141.0
Parental Education^c^, n, %						
High	45.0	23.1		109.0	47.3	
Intermediate	75.0	38.2		79.0	34.3	
Low	80.0	38.7		33.0	14.3	
Minor Neurosensory Dysfunction, n, %	37.0	18.5		13.0	5.6	
Minor Vision Loss or Corrected with Contact Lenses or Glasses	26.0	13.0		13.0	5.6	
Minor Hearing Loss or Corrected with Hearing Aids	5.0	2.5		0.0	0.0	
Spastic Unilateral Cerebral Palsy	6.0	3.0		0.0	0.0	

a Age of the very preterm children is not corrected for prematurity.^b^Adjusted for parental education. ^c^Highest of two parents. Low  =  primary education only or prevocational secondary education; intermediate  =  3-year secondary education or middle vocational education; high  =  higher professional, university training or PhD.

### Assessment of Mathematics and Attention

In the Netherlands, preschool starts at the child's fourth birthday and constitutes at least two years. Primary school starts with grade 1 in August for children who turn 6 years of age between October of the previous year and the following September. Mathematics in preschool and primary school were assessed using standardized tests that are part of the Dutch National Pupil Monitoring System.[38] A vast majority (±95%) of the Dutch schools uses this unique monitoring system for preschool and primary school pupils which enables teachers to monitor their pupils’ development in relation to both individual and peer development, at given moments during a school year, and over time.[38] Each derived raw test score is converted into an Ability score. Ability scores are collected throughout a school year and reflect progression in performance over time. Mathematical skills of children who were in preschool were assessed with the Numerical Reasoning test[39] which measures classifying, sorting, comparing, and counting of numbers or objects. Mathematical skills of children who were in primary school were assessed with the Mathematics test,[40] measuring the ability to solve written computational problems of addition, subtraction, multiplication, division, the notion of time, and use of money. For more information on these tests, please refer to www.cito.com.

Inattention in preschool children was rated by parents and teachers using the Attention Problems scale of the Child Behavior Checklist-Preschool [CBCL/1-5),[41] and the Teacher Report Form-Preschool (TRF/1-5),[41] respectively. Inattention in primary school children was rated by parents and teachers using the Attention Problems scale of the Child Behavior Checklist (CBCL/6-18),[42] and the Teacher Report Form (TRF/6-18),[42] respectively, as well as the Inattention subscale of the Disruptive Behavior Disorders parent and teacher rating scales (DBD/6-12).[43], [44]

To enhance reliability for these primary school questionnaires, we averaged scores on the parent DBD and CBCL attention scales. The same was done for the teacher DBD and TRF attention scales. Average scores were calculated for parent and teacher ratings separately, since interrater correlations were moderate (*r*<.52).[45]

### Assessment of Executive Function, Processing Speed, and IQ

EF’s that were measured included: 1) verbal fluency, measured in a test that required children to name as many examples of two specific categories: “animals” and “things you can eat or drink” within a 40-second time frame.[10] The dependent measure was the total number of correct responses. 2) Verbal working memory, assessed using the backwards condition of the Digit Span subtest of the Wechsler Intelligence Scale for Children-III (WISC-III).[46] Series of digits that were read by the examiner (one digit per second) were to be repeated in the reverse order. The dependent measure was the total number of correctly repeated series. 3) Visuospatial span was assessed with the Spatial Span subtest of the Cambridge Neuropsychological Testing Automated Battery (CANTAB).[47], [48] Children viewed a lighted sequence of squares and were required to reproduce the sequence by touching items on a touchscreen in the same order as originally illuminated. The dependent measure was the maximum span reached successfully. 4) Planning, assessed with the CANTAB subtest Stockings of Cambridge,[47], [48] which required children to solve problems by moving colored circles between three locations in a prescribed number of moves. The dependent measure was the number of problems solved. 5) Impulse control was measured with the Stop signal test[49] that required a child to respond as quickly and accurately as possible to a go-stimulus and to inhibit the response if a stop-stimulus was presented. The dependent measure was the number of commission errors that reflects the inability to inhibit an inappropriate response.[50]

Speed and inconsistency in speed were assessed using the correctly executed go-trials of the Stop Signal test,[49], [51] of which mean reaction time (MRT) and standard deviation of reaction times divided by MRT (SD of RT/MRT),[51], [52] were calculated.

IQ was measured with the subtests Vocabulary and Block Design of the WISC-III [46], or Wechsler Primary and Preschool Scale Intelligence-Revised (WPPSI-R)[53] (depending on the child's age). Subtest scores were used to calculate an estimated IQ, which correlates highly (.9 range) with full-scale IQ.[54]

### Procedure of Data Collection

Mathematical skills in preschool and in primary school were individually assessed by trained school staff. For very preterm children, completion of behavior questionnaires and assessment of EF and IQ took place at the Erasmus University Medical Centre Rotterdam Sophia Children's Hospital Rotterdam. Term children were assessed at their schools.

### Missing Data

Data on mathematics were available for 75.3% (n = 311) of the participating children. For the remaining children, data on mathematics were not available because they were either in special education (n = 24), or their school used a different pupil monitoring system (n = 30), or they were too young (n = 55) to be assessed with the mathematics test at the time of participation in our study.[5] Children of whom mathematical data were not available did not differ significantly from children of whom mathematical data were available in gestational age, birthweight, parental education (Fs<3.38, P_s_>.07), and gender (χ^2^_s_<1.10, P_s_>.30), except that they were younger (Fs>18.90, P_s_<.001).

Preschool parent rated attention was available for all children. Preschool teacher rated attention was available for 70.0% of the children. Gestational age, birthweight, parental education, and gender, did not differ between children with and without these teacher ratings (Fs<.68, P_s_>.41), although the latter group was on average 3 months younger (F = 5.03, P = .03). Primary school parent rated attention was available for 80.70% of the children and teacher rated attention was available for 74.10% of the children. Gestational age, birthweight, parental education, and gender, did not differ between children with and without these ratings (Fs<2.90, P_s_>.09), except that parents of children with teacher ratings available had a higher level of education, (F = 5.99, P = .02).

For dependent variables derived from the Verbal Fluency, Digit Span, and Stop Signal test, there was missing data (< 7.0%) which resulted from either examiner error or child noncompliance. These missing values were replaced by means of maximum likelihood estimation (Expectation Maximization).[55] Missing data for dependent variables of the Visuospatial Span and Stockings of Cambridge test (17.3% and 6.5%, respectively) resulted from hardware problems and were not replaced.

### Statistical Analyses

Whether poor mathematics and attention problems could be predicted from EF was examined using hierarchal linear regression analyses. These analyses were conducted separately for children in preschool and primary school. Raw scores were used in all analyses and P-values of <.05 (two-tailed) were considered statistically significant. R-square change values (δR^2^) of each step in the analyses were evaluated. Steps that did not reach the threshold for significance (P<.05) were not incorporated in further analyses. In step 1, the predictor group (very preterm versus term birth status) was entered, adjusted for grade (in case of analyses on mathematics[5]) or age (in case of analyses with attention ratings), gender, and most prestigious level of education of either the mother or the father. Interaction effects of group with grade or age, gender, and parental education, were tested. Steps 2 and 3 evaluated the effects of processing speed indices and IQ, respectively, and the two-way interaction effects with group. In step 4, EF dependent variables were entered using the forward selection procedure to select those EF independent variable(s) that maximized R-square, given the variables that were already selected. Results were expressed in terms of R-square (R^2^) and standardized regression coefficients (β) with values of.10, .30, and .50, referring to small, medium, and large effects, respectively.[56] All analyses were performed in PASW Statistics 18.0 (SPSS Inc, Chicago, IL, USA).

## Results

### Sample Characteristics

Table 1( presents sample characteristics for the very preterm and term group. As expected, very preterm children had a significantly lower mean gestational age P<.001), lower mean birth weight (P<.001), lower mean IQ (P<.001), lower mean level of parental education (P<.001), and more minor neurosensory dysfunction (P<.001) than controls. There were no significant group differences for age at assessment (P = .80), or sex (P = .30). Table 2 lists the perinatal characteristics of very preterm children.

**Table 2:** Perinatal Characteristics of Very Preterm Children.

Perinatal Characteristics	n	%
Intra Uterine Growth Retardation	47.0	23.3
Caesarian Section	120.0	60.0
Preeclampsia	65.0	32.5
Patent Ductus Arteriosus	84.0	42.0
Septicaemia	109.0	54.5
Necrotizing Enterocolitis Grade II/III	5.0	2.5
Respiratory Distress with Surfactant Treatment	131.0	65.5
Retinopathy of Prematurity Grade I/II/III	21.0/16.0/2.0	10.5/8.0/1.0
Intra-Ventricular Hemorrhage Grade I/II/III/IV	17.0/25.0/8.0/2.0	8.5/12.5/4.0/1.0
Oxygen Dependence at 6 Weeks Corrected Age	11.0	5.4
Prenatal Steroids (Celestone)	141.0	70.5
Postnatal Steroids (Dexamethasone)	35.0	17.3
Dopram	62.0	31.0
Duration of Assisted Ventilation, mean, SD, days	9.1	0.2
Duration of Stay on Neonatal Intensive Care, mean, SD, days	43.0	36.8

Intra uterine growth retardation is defined as an SDS score of -2.0*SD* below expectation for gestational age. Septicaemia was defined as a positive blood culture. Necrotizing enterocolitis was defined according to criteria given by Bell et al. Respiratory distress was defined as requiring assisted ventilation.

### Associations Between Processing Speed Indices, IQ, EF, Mathematics, and Attention

Table 3 displays the summary of the hierarchical linear regression analyses using IQ, speed indices, and EF as predictors for mathematics and attention problems.

**Table 3:** Summary of Hierarchical Linear Regression Analyses Using IQ, Speed Indices, and Executive Function, as Predictors for Mathematics and Attention Problems.

Step	Predictor	ΔR^2^	P	β	P
	**Preschool**				
	**Mathematics**				
1	Group^a^	**.34**	**<.001**	**−.35**	**.006**
2	Group^a^	**.46**	**.007**	−.17	.21
	IQ			**.35**	**.007**
**Parent**	**Rated Attention Problems**				
1	Group^a^	**.13**	**.006**	**.32**	**.004**
4	Group^a^	**.18**	**.04**	**.26**	**.03**
	Impulse Control			**.25**	**.04**
**Teacher**	**Rated Attention Problems**				
1	Group^a^	**.20**	**.003**	**.41**	**.003**
	**Primary School**				
	**Mathematics**				
1	Group^a^	**.80**	**<.001**	**−.11**	**.01**
4	Group^a^	**.84**	**<.001**	−.04	.16
	Speed Indices			−.05	.10
	IQ			**.16**	**<.001**
	Visuospatial Span			**.13**	**<.001**
**Parent**	**Rated Attention Problems**				
1	Group^a^	**.21**	**<.001**	**.23**	**<.001**
4	Group^a^			**.15**	**.04**
	IQ			−.14	.06
	Visuospatial Span			**−.16**	**.04**
**Teacher**	**Rated Attention Problems**				
1	Group^b^	**.25**	**.01**	**.30**	**<.001**
	Group*Gender			**−.23**	**.005**
4	Group^b^	**.38**	**.009**	**.18**	**.01**
	Group*Gender			**−.18**	**.03**
	IQ			−.005	.96
	Group*IQ			**−.24**	**.02**
	Visuospatial Span			**−.19**	**.009**
	Impulse Control			**.19**	**.005**

Significant associations (*P*<.05) are shown in bold type.^a^Effects of group have been adjusted for grade or age, gender, and most prestigious level of parental education.^b^Effects of group have been adjusted for grade or age, and most prestigious level of parental education.

In preschool, very preterm versus term birth status (i.e. group) significantly predicted poor mathematics and parent and teacher rated inattention. Step 2 and 3 yielded significant effects of IQ on mathematics. Processing speed indices were not significantly predictive for mathematical nor for attention problems (ΔR^2^<.04, β_s_<−.21, P_s_>.15). In Step 4, impulse control significantly predicted parent rated attention problems over and above speed and IQ, but EFs did not significantly predict mathematical problems nor teacher rated attention problems (β_s_<−.29, P_s_>.10).

In primary school, group significantly predicted poor mathematics. Step 2 and 3 yielded significant effects of processing speed and IQ on mathematics, although effects of processing speed disappeared (β = −.05, P = .10), with EFs entered into the analyses in Step 4. Of the EFs entered in Step 4, poor visuospatial span significantly predicted poor mathematics. Group also significantly predicted parent rated and teacher rated inattention, and a significant interaction between group and gender (ΔR^2^ = .03, β = −.23, P = .005) indicated that very preterm boys had the highest teacher ratings of inattention. Step 2 and 3 yielded significant effects of low IQ on parent and teacher rated inattention (β_s_>−.18, P_s_<.02). A significant interaction with group (ΔR^2^ = .03, β = −.26, P = .01) indicated that the association between IQ and teacher rated inattention was significantly stronger for very preterm than for term children. Processing speed indices did not significantly predict parent nor teacher rated inattention (ΔR^2^<.02, β_s_<.13, P_s_>.08). Of the EFs, entered in Step 4, poor verbal working memory and visuospatial span significantly predicted parent rated attention problems and poor visuospatial span and impulse control significantly predicted teacher rated attention problems. None of the remaining interactions with group were significant.

## Discussion

This study compared 200 very preterm children to 230 term control children on measures of mathematics and parent and teacher ratings of attention with the main hypothesis of the study being that poor EF would account, over and above response speed indices and IQ, for problems in mathematics and symptoms of inattention in very preterm children. Since such mathematical and attention problems are two of the most commonly reported problem areas in this population,[3] a better understanding of the underlying neurocognitive impairments will contribute to prediction of these problems and open up possibilities for appropriate intervention programs. Analyses were conducted separately for two subsamples: children in preschool and children in primary school.

Results confirmed that very preterm children performed worse than term peers on all measures of mathematics and attention. Poor mathematics in preschool was fully accounted for by IQ. In primary school both IQ and EF accounted for group differences in mathematics, suggesting that in primary school, mathematical problems become increasingly complex and demanding and appeal to a higher level of neurocognitive abilities. The strong impact of IQ on mathematics reflects visuospatial as well as verbal abilities being important requisites for mathematical achievement.[7], [57]–[59] This study also showed the unique contribution of the EF visuospatial span to very preterm children's poor mathematical achievement over and above that of IQ, showing that a good understanding of visuospatial relations as well as the ability to manipulate this material in working memory is critical to very preterm children's mathematical achievement in primary school.

The EF impulse control explained unique variance in parent rated inattention in preschool, which corresponds with the view that impulse control deficits underlie inattentiveness in young children,[60] and may be a precursor for later ADHD.[8], [61] Future studies with longitudinal data should, however, address this issue, given our finding that these relationships were not observed for teacher ratings, and the fact that inattentiveness in preschool may, for instance, also reflect immaturity in behavioral adjustment, rather than ’true’ attention deficits in isolation.[62] In the normal population, attention problems at preschool age appear to be persistent in only 5.0*%* of children.[63], [64]

In primary school, both IQ as well as EF explained unique variance in parent and teacher rated inattention. In addition, gender interacted with group indicating that the excess in attention problems as rated by teachers was mainly found in very preterm boys. IQ also interacted with group in the prediction of teacher rated attention problems in primary school, such that IQ showed stronger effects on inattention in very preterm than in term infants. Visuospatial span explained unique variance in parent as well as in teacher rated inattention, though impulse control only explained unique variance in teacher rated inattention, findings that converge with the few earlier studies on this issue.[65]–[67] The inconsistency between to which extent parent and teacher ratings are associated with EF task performance has been observed previously and may be related to the fact that teachers generally are more optimal informants for attention problems.[68], [69] A classroom situation in primary school, however, may also be more demanding, thereby more heavily appealing to executive skills than a home situation.[70] Our results confirm strong associations between poor visuospatial skills, impulse control, and inattention, both subserved by fronto-striatal and frontal-parietal networks,[71], [72] which have also been described in ADHD children, and converge with findings of abnormalities in these neural structures in the very preterm population.[73], [74]

Processing speed indices did not uniquely contribute to mathematics nor to attention problems. Though,‘lower-order’ speed is impaired in very preterm children, it does not capture contributions of ‘higher-order’ executive processes.[32], [75] Comparison of our findings to those of studies that did find a significant effect of speed[20], [22] is precluded because these studies have employed speed measures that require greater cognitive efforts than our measures do or depend on fine-motor skills,[76], [77] which have been described as affected in very preterm birth survivors.

In our study, effects of EF were calculated while adjusting for IQ, whereas in earlier studies, e.g.[22], [23], effects of EF were compared to those of IQ. Because EF and IQ share some variance (r_s_<.30)[35], the impact of EF was smaller in our study than in these earlier studies. Our approach, however, enabled calculation of the unique contribution of EF over and above that of IQ. The wide age range of participating children enabled to examine associations both for preschool as well as for primary school children; however, the cross-sectional design of the study limited the possibility to compare effects over time. In preschool, EF is presumed to be not yet as fractionated as it is at middle school age.[78] A longitudinal dataset would have enabled to perform growth curve analyses to examine the emergence of fractionation of EF in relation to the development of mathematical and attentional skills. It should also be noted that mathematical skills were assessed by school staff which may have biased the test scores. Strengths of this study include the overall large sample size and selection of appropriate measures of EF. Inclusion of measures of speed is another positive feature of the study, especially given the views of some researchers that processing speed may be a critical factor in accounting for the effects of preterm birth on mathematics.[22], [23], [75]

## Conclusion

Very preterm birth is associated with deficits in mathematics and symptoms of inattention. This study showed that impaired EF was, over and above impaired IQ, an important predictor for these adverse outcomes. Given the increasing body of research[20], [23], [32], [70], [75], [79] proving that EF is fruitful in identifying those very preterm children at risk for mathematical deficits and attention problems, an important theme for future research could be the development of intervention programmes directed at specific improvement of EF in very preterm children at early ages. Intervention techniques proven to have significant effects include computerized cognitive training programs[80]–[83] EF has also been shown to be highly sensitive to effects of methylphenidate.[51] In addition, the practice of neonatal follow-up care may expand their conventional IQ assessments with EF and provide long-term follow-up care since the (pre)frontal cortex subserving EF develops rapidly up to young adulthood[84], [85] in order to identify and monitor those children in need for support.

## Supporting Information

Table S1 **Correlation Coefficients for Associations Between IQ, Processing Speed Indices, EF, Mathematics, and Attention Ratings in Preschool and Primary School.**
(DOC)
