# Supplementary material for: Executive Function and IQ Predict Mathematical and Attention Problems in Very Preterm Children
Source: PLoS One. 2013 Feb 4;8(2):e55994. doi: 10.1371/journal.pone.0055994 (PMC3563540; doi:10.1371/journal.pone.0055994)
Supplement: Table S1 — Correlation Coefficients for Associations Between IQ, Processing Speed Indices, EF, Mathematics, and Attention Ratings in Preschool and Primary School. (DOC) [file pone.0055994.s001.doc]

Appendix Correlation Coefficients for Associations Between IQ, Processing Speed Indices, EF, Mathematics, and Attention Ratings in Preschool and Primary School

|  | | IQ | Processing Speed Indices | | Executive Function | | | | | Mathematics | | Attention Ratings | | |
| --- | --- | --- | --- | --- | --- | --- | --- | --- | --- | --- | --- | --- | --- | --- |
|  | | 1 | 2 | 3 | 4 | 5 | 6 | 7 | 8 | 9 | 10 | 11 | 12 | 13 |
| 1 | IQ (n = 400) |  |  |  |  |  |  |  |  |  |  |  |  |  |
| 2 | Mean Processing Speed (n = 375) | **-.16** |  |  |  |  |  |  |  |  |  |  |  |  |
| 3 | Fluctuations in Processing Speed (n = 375) | **-.22** | **.49** |  |  |  |  |  |  |  |  |  |  |  |
| 4 | Verbal Fluency (n = 400) | **.28** | **-.50** | **-.35** |  |  |  |  |  |  |  |  |  |  |
| 5 | Verbal Working Memory (n = 400) | **.30** | **-.41** | **-.33** | **.67** |  |  |  |  |  |  |  |  |  |
| 6 | Visuospatial Span (n = 335) | **.25** | **-.47** | **-.40** | **.63** | **.62** |  |  |  |  |  |  |  |  |
| 7 | Planning (n = 377) | **.17** | **-.37** | **-.27** | **.48** | **.47** | **.48** |  |  |  |  |  |  |  |
| 8 | Impulse Control (n = 375) | **-.14** | **.33** | **.52** | **-.45** | **-.48** | **-.41** | **-.37** |  |  |  |  |  |  |
| 9 | Preschool Mathematics (n = 55) | **.42** | -.25 | **-.31** | **.38** | **.47** | .29 | **.29** | -.21 |  |  |  |  |  |
| 10 | Primary school Mathematics (n = 256) | **.32** | **-.41** | **-.34** | **.58** | **.53** | **.59** | **.42** | **-.33** | NA |  |  |  |  |
| 11 | Preschool Parent Rated Attention (n = 117) | **-.20** | .15 | **.26** | **-.27** | -.14 | -.16 | -.03 | **.29** | -.20 | NA |  |  |  |
| 12 | Preschool Teacher Rated Attention (n = 73) | **-.24** | .19 | .00 | -.22 | -.22 | **-.30** | -.23 | .09 | **-.47** | NA | **.33** |  |  |
| 13 | Primary School Parent Rated Attention(n = 248) | **-.30** | .04 | **.21** | **-.14** | **-.18** | **-.18** | -.10 | **.16** | NA | **-.17** | NA | NA |  |
| 14 | Primary School Teacher Rated Attention (n = 233) | **-.36** | **.15** | **.27** | **-.19** | **-.25** | **-.25** | **-.21** | **.32** | NA | **-.34** | NA | NA | **.53** |

Significant (P < .05) correlation coefficients are shown in bold type. NA = Not Available.
